# TL1A morphological shifts and inflammatory heterogeneity in rhinosinusitis and allergic rhinitis: a hypothesis-driven review

**DOI:** 10.3389/fimmu.2026.1948935

**Published:** 2026-10-07

**Authors:** Tianwei Pei, Xiang Li

**Affiliations:** Department of Anesthesiology, Dalian No.3 People’s Hospital, Dalian, Liaoning, China

**Keywords:** chronic rhinosinusitis, endotypic heterogeneity, macrophage, tumor necrosis factor-like ligand 1A (TL1A), type 2 inflammation

## Abstract

Allergic rhinitis (AR) and chronic rhinosinusitis (CRS) are heterogeneous upper-airway inflammatory disorders in which type 2 inflammation is common but does not fully explain all clinical endotypes or treatment responses. This review synthesizes evidence on tumor necrosis factor-like ligand 1A (TL1A) and considers whether its membrane-bound (mTL1A) and soluble (sTL1A) forms could connect epithelial injury, macrophage activation, and mixed inflammatory networks. Evidence from intestinal and lower-airway models supports TL1A/DR3 signaling in several immune contexts, while sinonasal studies provide only limited, cell-specific evidence and do not yet establish local TL1A abundance, shedding dynamics, or a complete macrophage-centered feedback loop. We therefore propose a testable model in which mTL1A may support contact-dependent signaling, whereas epithelial stress and protease-mediated shedding may generate soluble TL1A with broader paracrine activity. In this framework, TL1A could cooperate with IL-33, TSLP, and IL-25 and might activate macrophage-dependent relays involving CXCL8/IL-8 and IL-1beta. Because neutrophils are generally considered indirect targets of TL1A/DR3 signaling, such relays could contribute to mixed or treatment-refractory inflammation without implying that TL1A alone is sufficient. The model provides a conceptual framework for integrating endotypic heterogeneity and identifying testable biomarkers and therapeutic hypotheses, but direct validation in human sinonasal tissue, nasal lavage fluid, and relevant experimental models remains necessary.

## Graphical Abstract

**Figure:**
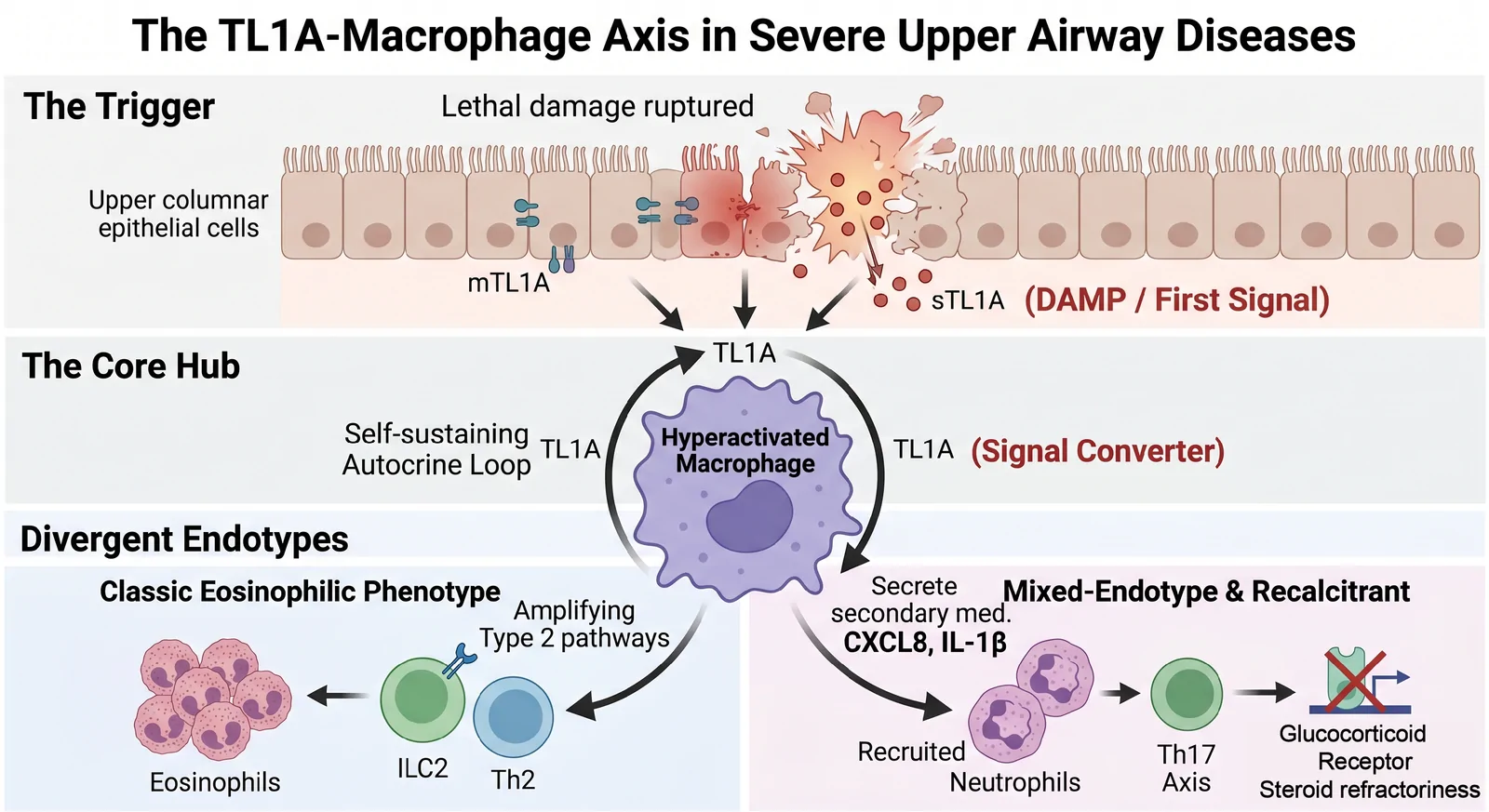
The proposed TL1A-macrophage framework links epithelial stress, receptor-bearing immune cells, macrophage feedback, and mixed inflammatory mediators. Evidence is strongest in selected intestinal and lower-airway contexts, while direct CRS/AR validation of local TL1A forms, cell sources, and causal feedback remains necessary.

## 1 Introduction

Upper airway inflammatory diseases, particularly allergic rhinitis (AR) and chronic rhinosinusitis (CRS), impose a substantial global burden and comprise clinically and biologically heterogeneous disorders (1). Type 2 inflammation, involving ILC2s, Th2 cells, eosinophils, and epithelial alarmins, is prominent in many patients, but mixed type 2/type 17 patterns, neutrophilic inflammation, tissue remodeling, and variable treatment responses are also well documented (2–6). This heterogeneity limits the assumption that one downstream cytokine or one cellular compartment explains the full disease spectrum. Although biologics directed at type 2 pathways can be effective, responses are not uniform, particularly in difficult-to-treat or recurrent disease (7–11). Alternative or interacting upstream influences include epithelial-barrier dysfunction, eicosanoid imbalance, fibroblast remodeling, and microbiome dysbiosis, all of which may shape the inflammatory context in which cytokine networks operate (12–16).

TL1A, encoded by TNFSF15 and signaling mainly through death receptor 3 (DR3), is an attractive candidate connector because its membrane-bound and soluble forms can support different spatial modes of communication. Strong mechanistic evidence from intestinal inflammation and lower-airway disease indicates that TL1A/DR3 can regulate lymphoid activation, tissue remodeling, and inflammatory amplification (17–22). Recent airway work further supports epithelial TL1A as an alarmin that can cooperate with IL-33 in allergic inflammation (23, 24). These findings provide biological plausibility, but they do not establish that the same source, shedding process, receptor distribution, or feedback architecture operates in human CRS or AR. Direct sinonasal measurements of mTL1A and sTL1A, especially in nasal polyps, inferior turbinate mucosa, and nasal lavage fluid, remain insufficient to define a reproducible morphological switch. Accordingly, this review treats the TL1A-macrophage model as a hypothesis to be tested rather than as an established universal mechanism. We distinguish evidence obtained in sinonasal samples from inferences derived from other mucosal or airway systems and identify predictions that could be examined in patient-derived tissues, lavage samples, organoids, and relevant animal models (17). The relationship among the mechanisms and their evidence boundaries is summarized in **Table 1**; **Figure 1**.

**Table 1:** Proposed TL1A-related mechanisms and current evidence boundaries in upper-airway inflammatory heterogeneity.

Dimension/feature	Traditional pathological profile & biologic limitations	Proposed TL1A-related mechanisms and potential therapeutic implications	References
Epidemiological & Clinical Burden	High global prevalence of AR and CRS across diverse demographics; presents with debilitating symptoms that profoundly impair quality of life, sleep, and daily productivity.	TL1A is a candidate upstream regulator within complex networks; its relevance to CRS/AR remains to be determined.	(1–4)
Inflammatory Cascades	Classically orchestrated by T helper 2 (Th2) cells and type 2 innate lymphoid cells (ILC2s) via downstream effector cytokine pathways.	May bridge multiple inflammatory pathways, based mainly on IBD and lower-airway evidence; direct upper-airway validation is limited.	(5–7, 14)
Tissue & Mucosal Remodeling	Characterized by subepithelial fibrosis, goblet cell hyperplasia, robust tissue eosinophilia, and architectural distortion leading to nasal polyp formation.	Could contribute to mucosal remodeling through proposed structural and cellular transitions; direct causal evidence in sinonasal tissue is lacking.	(5–8)
Therapeutic Challenges & Targets	Suboptimal clinical efficacy of targeted monoclonal antibodies (e.g., anti-IL-4/IL-5) in patients characterized by mixed inflammatory infiltrates and endotypic heterogeneity.	Could represent an upstream candidate in refractory disease, but clinical efficacy in CRS/AR has not been established.	(9–13)
Cellular Cross-Talk & Loops	Traditional targets focus on linear pathways, failing to control refractory cases due to the independence and persistence of mixed inflammatory networks.	A putative macrophage-mediated autocrine/paracrine network that may connect lymphoid and myeloid responses.	(12, 14)
Direct sinonasal evidence	Human sinonasal evidence is limited to selected receptor-competent polyp-derived cells and selected TNFSF measurements; these findings should not be conflated with asthma or IBD data.	DR3+ memory CD4+ T cells and TL1A responsiveness have been reported in nasal polyp-derived cells, but reproducible mTL1A/sTL1A measurements in nasal polyps, inferior turbinate mucosa, nasal lavage fluid, or matched clinical specimens, as well as a complete macrophage loop, remain unconfirmed.	(25–27)

**Figure 1:**
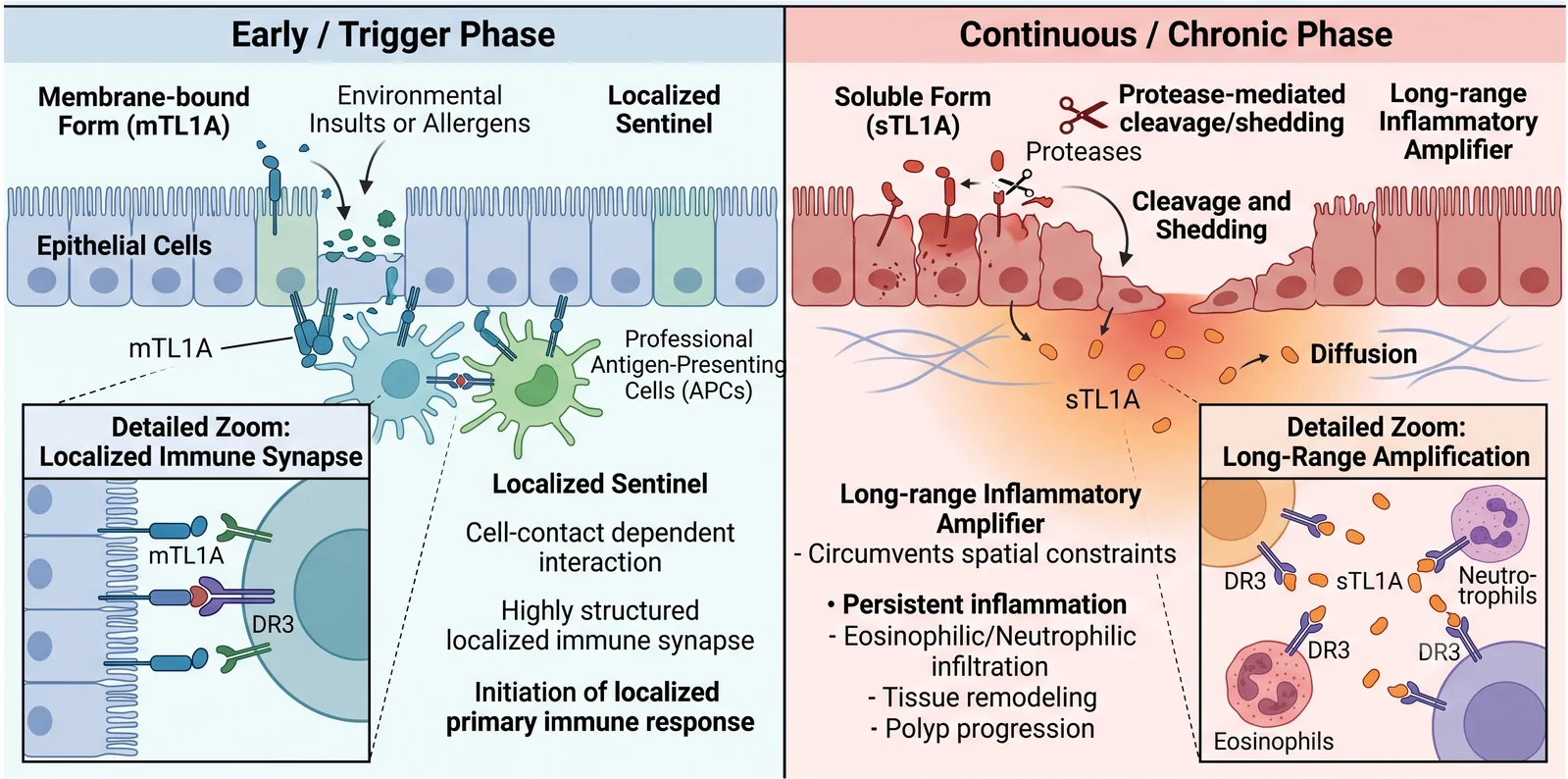
Proposed spatiotemporal framework for TL1A morphology in upper-airway inflammation. (Left Panel) The figure proposes that membrane-bound TL1A (mTL1A) could support localized contact-dependent signaling after environmental or allergen exposure. (Right Panel) Protease-mediated cleavage could generate soluble TL1A (sTL1A) with broader paracrine activity during persistent inflammation. These spatial assignments and the proposed shift from mTL1A to sTL1A are supported mainly by other mucosal or airway systems; they are not intended to depict a fixed chronological sequence, and direct CRS/AR validation of local abundance, timing, and targets is still needed.

In summary, this review maps a proposed relationship between TL1A morphology, epithelial stress, macrophage activity, and inflammatory heterogeneity. It explicitly separates direct upper-airway observations from evidence extrapolated from intestinal and lower-airway systems, considers alternative upstream drivers, and identifies testable predictions concerning local TL1A abundance, shedding, DR3 distribution, CXCL8/IL-8 signaling, and therapeutic delivery (**Table 1**).

## 2 Morphological shifts of TL1A and Its spatiotemporal evolution across disease stages

The pathogenic versatility of TL1A in upper airway diseases is intrinsically linked to its structural dynamics. To bridge its molecular characteristics with clinical disease progression, this section examines the principal morphological states of TL1A and maps their possible functional roles across different disease phases and experimental contexts (**Figure 1**).

### 2.1 Molecular conformations and spatial distribution

The biological pleiotropy of TL1A is related to its two principal molecular forms: membrane-bound TL1A (mTL1A) and soluble TL1A (sTL1A) (18). The structural distinction is established in several mucosal and lower-airway contexts, where an intact transmembrane precursor can mediate contact-dependent signaling and proteolytic cleavage can release an extracellular ligand (19, 20, 28, 29). However, the relative abundance, cellular sources, and spatial distribution of these forms in human CRS and AR have not been systematically defined. The framework used here therefore treats the mTL1A-to-sTL1A transition as a proposed organizing concept rather than a demonstrated sinonasal sequence (**Table 2**; **Figure 1**).

**Table 2:** Proposed comparative characteristics of TL1A morphological variants in upper-airway inflammation.

Characteristic	Proposed membrane-bound TL1A (mTL1A)	Proposed soluble TL1A (sTL1A)	References
Structural Conformation	Intact transmembrane precursor	Cleaved extracellular form	(15–19)
Mucosal Distribution	Cell-surface localization described in other tissues; sinonasal distribution not established	Extracellular diffusion after shedding described in other tissues; sinonasal levels not established	(16–19)
Mode of Interaction	Contact-dependent signaling is supported in experimental models	Long-range signaling is supported in other tissues; upper-airway relevance remains hypothetical	(18, 21, 22, 28)
Disease Phase	Proposed early/trigger phase; not established in CRS/AR	Proposed chronic/continuous phase; not established in CRS/AR	(20, 23, 24)
Primary Function	Candidate local alarm signal	Putative inflammatory amplifier	(22, 28)
Pathological Outcome	Potential initiation of localized responses	Potential contribution to persistent infiltration/remodeling	(22, 29, 30)

Direct sinonasal evidence remains limited and should be distinguished from evidence derived from asthma, lower-airway, or gastrointestinal disease. Holmkvist et al. detected IL-18Ralpha+DR3+ memory CD4+ T cells in human nasal polyps and demonstrated their ex vivo responsiveness to TL1A, supporting receptor competence in a subset of polyp-derived cells rather than proving local TL1A production (30). This study did not quantify TL1A protein or distinguish mTL1A from sTL1A in nasal polyp tissue, nasal lavage fluid, or inferior turbinate mucosa. In a CRSwNP survey of TNFSF members, the TNFSF15 signal was not elevated in nasal polyp tissue, whereas TNFSF11 was increased and TSLP enhanced TNFSF11-induced IL-13 production by ILC2s (31, 32). The available sinonasal studies also do not yet provide a consistent, paired, cell-resolved comparison across nasal polyps, inferior turbinate mucosa, nasal lavage fluid, blood or serum, and other clinical specimens. Thus, current data do not establish a reproducible sTL1A/mTL1A gradient, local shedding rate, or macrophage-specific TL1A source in human CRS or AR. These uncertainties are central to the hypothesis-driven model developed below.

### 2.2 The early trigger phase: mTL1A as a localized sentinel

The progression of upper-airway inflammation may involve different TL1A signaling geometries, beginning with a putative contact-dependent phase (33). In experimental systems, mTL1A on an activated cell can engage DR3 on a neighboring receptor-positive cell and restrict signaling to a local immune synapse (21, 22, 29). Whether epithelial cells, macrophages, or other sinonasal cells provide the dominant mTL1A source in AR or CRS, and whether this interaction is required *in vivo*, remains unresolved. The term “early trigger” is therefore used as a testable model for one possible disease context, not as proof of a universal initiating event.

### 2.3 The chronic phase: sTL1A as a putative long-range amplifier

As inflammation persists, protease activity, barrier disruption, and remodeling could favor release of soluble TL1A from cell-surface pools (34). In other tissues, ADAM17 and matrix metalloproteinases can participate in cytokine shedding, and sTL1A can diffuse beyond the producing cell (19, 20, 35). In the sinonasal mucosa, however, the relevant proteases, shedding kinetics, concentration range, and target-cell responses have not been established. sTL1A could therefore act as a long-range inflammatory amplifier by engaging DR3-positive lymphoid or myeloid populations and by interacting with downstream chemokine and remodeling programs, but the extent to which this occurs in CRS or AR remains a hypothesis (22, 36–38).

In summary, the transition from mTL1A to sTL1A is a useful hypothesis for organizing spatial and temporal TL1A biology, but it has not been established as a universal molecular switch in AR or CRS. Testing this model will require paired measurement of membrane-associated and soluble TL1A in nasal polyp tissue, inferior turbinate mucosa, nasal lavage fluid, and relevant cell cultures, together with direct assessment of shedding and DR3-dependent responses.

## 3 Potential early-trigger network: epithelial injury-associated TL1A release and macrophage activation

To consider how epithelial injury might be connected to early myeloid activation, it is useful to distinguish possible lytic-release routes from chronic cytokine shedding. Pyroptosis, necroptosis, and other forms of epithelial stress can release alarmins and damage-associated molecular patterns in airway tissues (39). TL1A could participate in such a response, but the timing, cellular source, and relative contribution of TL1A during CRS or AR exacerbations have not been directly established. **Figure 2** therefore presents a plausible early-release route and its experimental gaps rather than a confirmed sequence.

**Figure 2:**
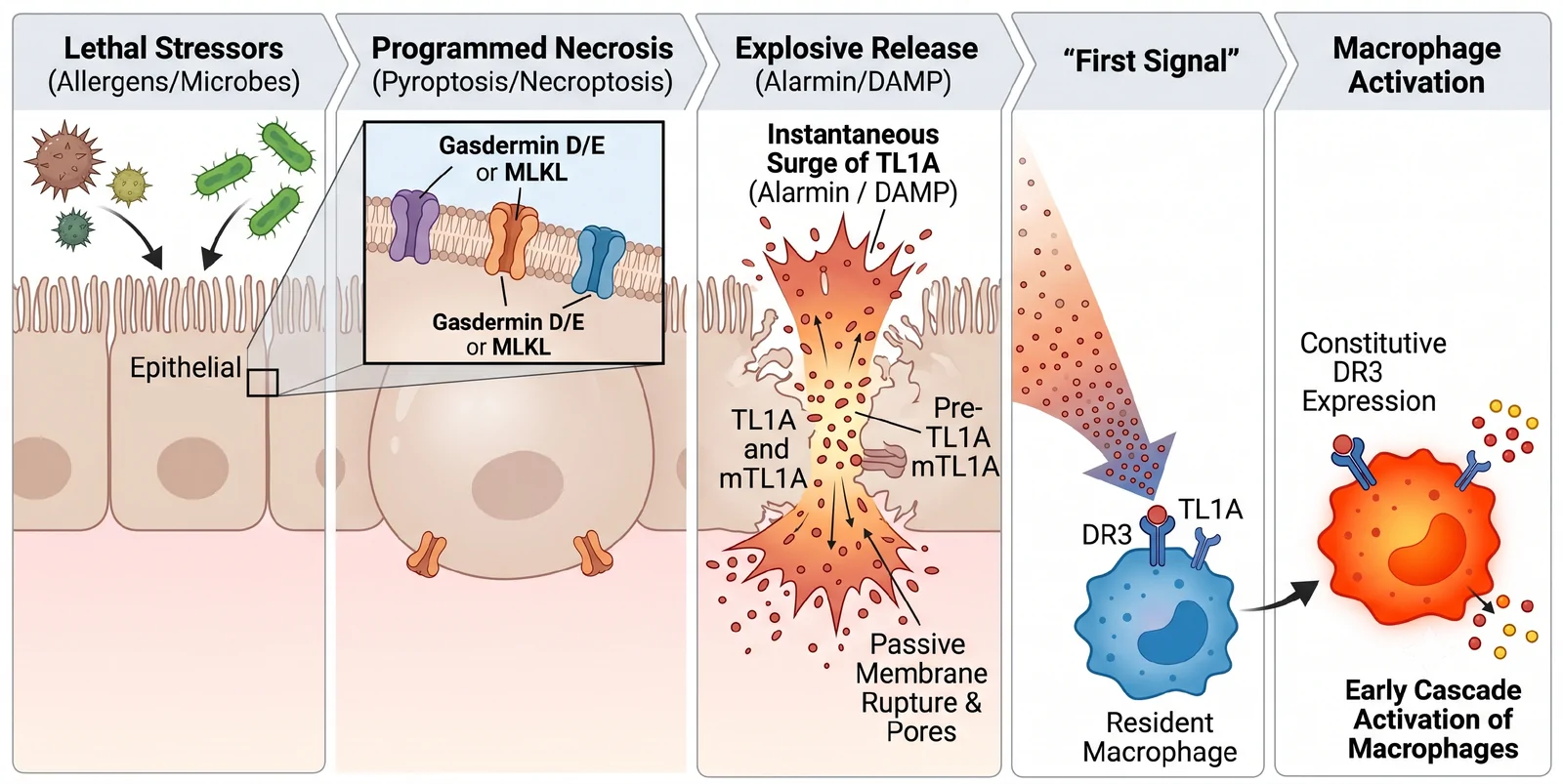
A proposed route linking epithelial injury-associated TL1A release with macrophage activation. Lethal stressors (e.g., severe allergens or pathogenic microbes) may induce programmed necrosis or other lytic injury in sinonasal epithelial cells, with Gasdermin D/E and MLKL representing candidate execution pathways (39). Loss of membrane integrity could alter the local TL1A pool and engage DR3-positive macrophages, but the timing, source, and magnitude of TL1A release, and its necessity for early macrophage activation, have not been established in CRS or AR. The figure is a conceptual hypothesis rather than a validated sequence.

### 3.1 Lethal epithelial stress and programmed necrosis

In contrast to gradual protease-dependent shedding, severe epithelial injury can cause loss of membrane integrity and release of intracellular or membrane-associated mediators (20, 39). Gasdermin-mediated pyroptosis and MLKL-associated necroptosis are candidate mechanisms that could alter the local alarmin environment (40–42). Whether TL1A is pre-stored in sinonasal epithelial cells, generated during injury, or primarily supplied by neighboring immune cells is unknown. Thus, a lytic event may provide one route for rapid TL1A mobilization, but it should not be interpreted as evidence that TL1A is the defining or necessary epithelial death signal in CRS or AR (43–47).

### 3.2 Rapid release of TL1A as an alarmin (DAMP)

The abrupt rupture of an injured epithelial cell can passively release intracellular contents and membrane-associated factors into the submucosal space (48, 49). TL1A could be mobilized directly or indirectly during this process, depending on its subcellular localization and proteolytic processing. The proposed model does not assume a pre-existing intracellular store or a fixed concentration surge in sinonasal tissue; these points require biochemical and spatial validation. In practice, the local response would also reflect co-released alarmins, extracellular-matrix fragments, microbial products, and eicosanoids, which may act in parallel with TL1A (50–55).

### 3.3 Candidate early signal: macrophage activation

A rapid TL1A signal from an injured epithelial compartment could engage DR3-positive macrophages and other receptor-bearing cells, thereby contributing to early myeloid activation (56, 57). Evidence from other mucosal and airway systems supports TL1A responsiveness in selected immune populations, but it does not show that epithelial TL1A is the primary or necessary initiating signal in human CRS or AR. The macrophage response may instead require cooperation with IL-33, TSLP, IL-25, microbial ligands, or tissue-derived danger signals. These alternatives can be tested by time-resolved sampling and receptor-specific perturbation in sinonasal models (58–64).

To clarify the distinct biological logic between these release mechanisms, **Figure 3** contrasts gradual protease-mediated shedding with an acute injury-associated release route. These are two plausible, non-exclusive mechanisms rather than proven sequential stages. Whether they coexist in chronic sinonasal tissue, represent different phases of disease, or contribute mainly during acute exacerbation remains unresolved. The figure is intended to organize testable possibilities, not to establish a fixed chronic-versus-acute chronology.

**Figure 3:**
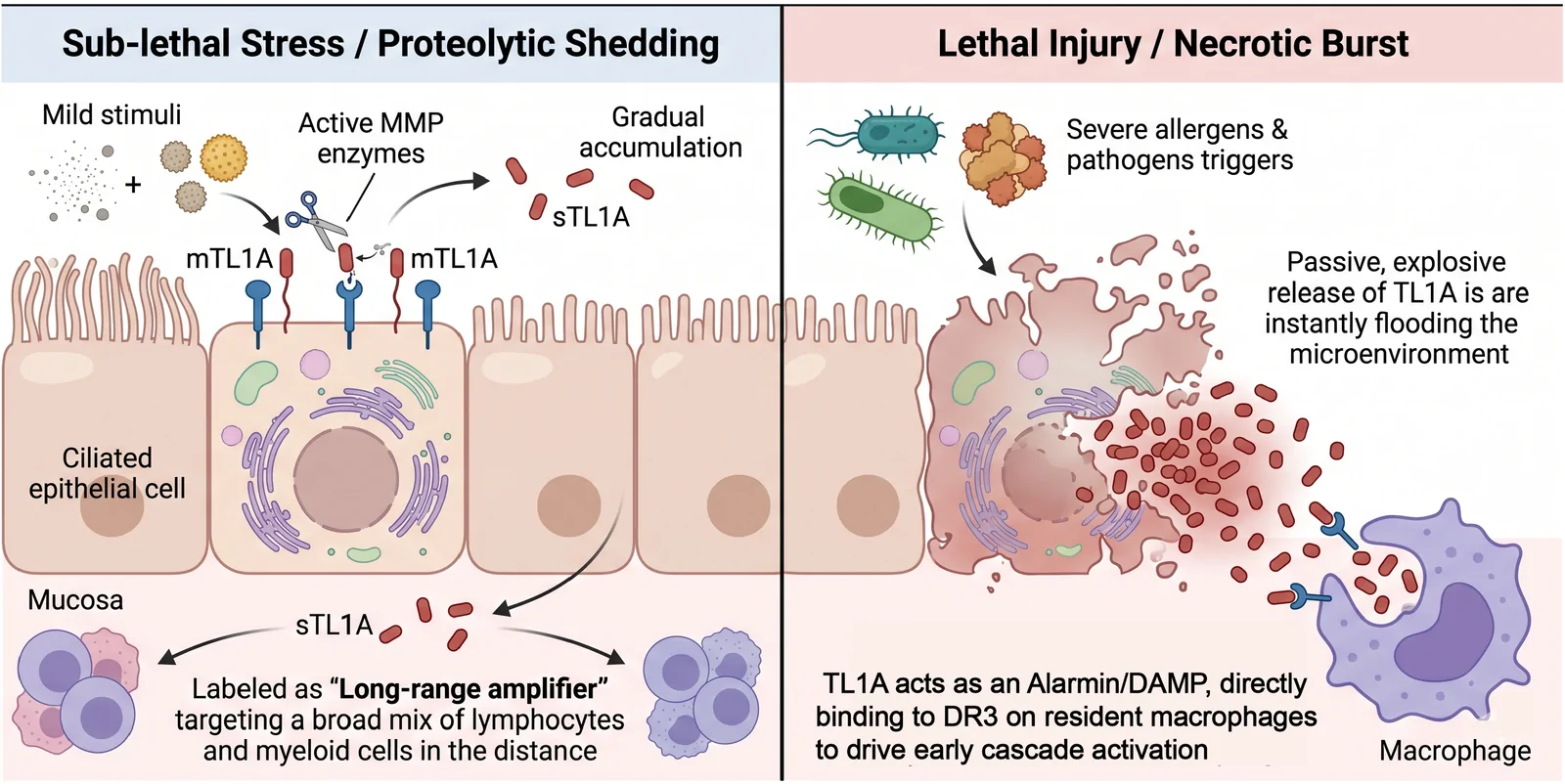
Conceptual routes of TL1A mobilization in upper-airway epithelium. Under sub-lethal stress, protease-mediated cleavage may gradually shed mTL1A and generate sTL1A, whereas lethal injury may provide a distinct rapid-release route. These mechanisms are plausible and non-exclusive; the figure does not establish that they occur sequentially, coexist throughout chronic sinonasal disease, or represent fixed chronic and acute stages. Their relative importance in AR, CRS, and acute exacerbations remains to be tested.

## 4 Candidate mechanism: a macrophage autocrine/paracrine remodeling loop

If epithelial injury or another local stimulus activates DR3-positive macrophages, the response could transition from a short-lived alarm to a self-reinforcing inflammatory state. A macrophage-centered loop is biologically plausible because macrophages can both respond to and produce inflammatory mediators (65). Nevertheless, the proposed independence of this loop from continuing epithelial injury, and its relevance to human CRS or AR, remain to be demonstrated by cell-specific measurements and perturbation studies. **Figure 4**; **Table 3** summarize the proposed cellular relationships and distinguish them from established epithelial-alarmin biology.

**Figure 4:**
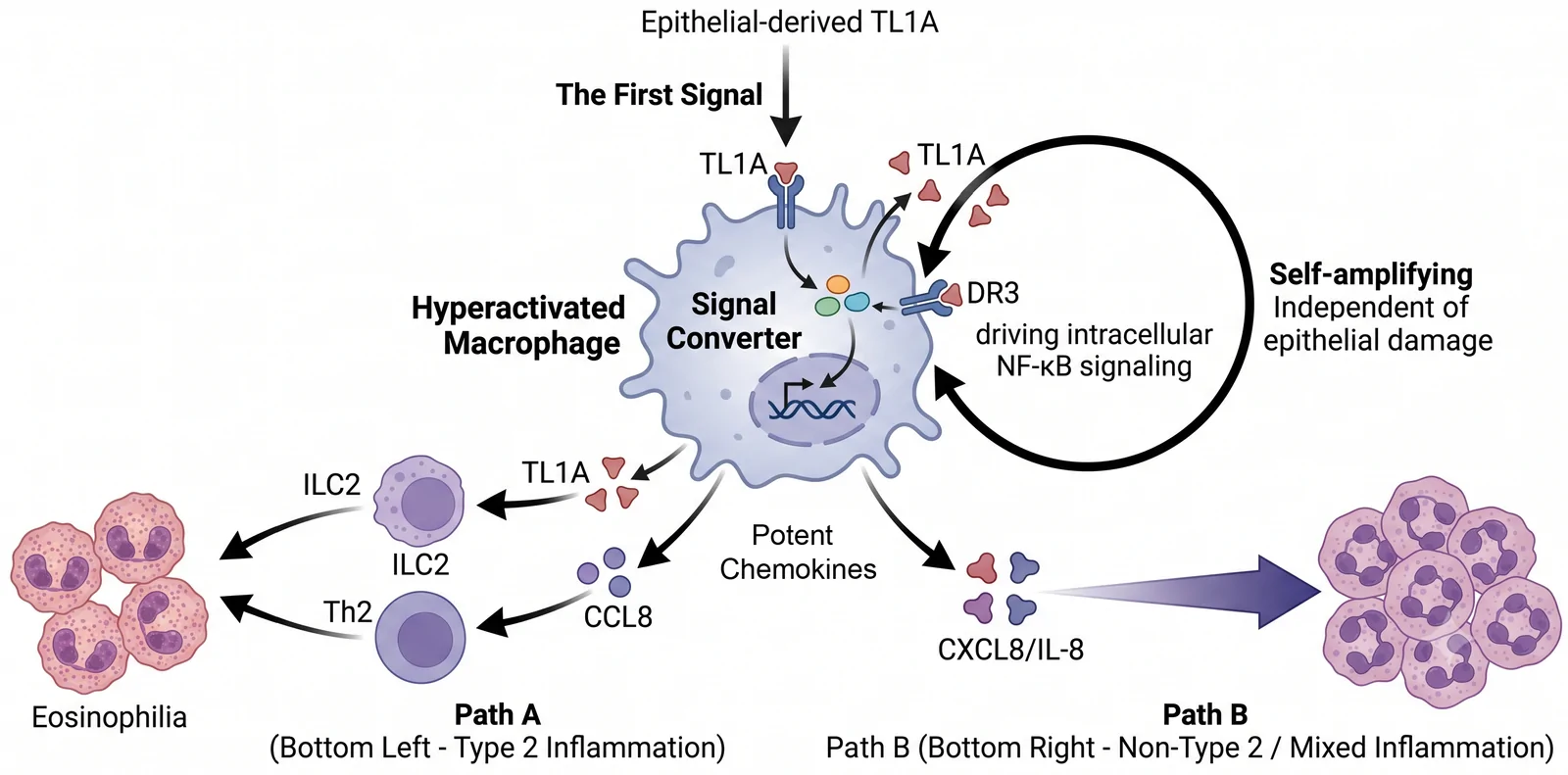
Proposed macrophage-mediated autocrine and paracrine network. If epithelial-derived TL1A activates a macrophage, that cell could produce additional TL1A and other mediators, creating a putative feedback loop. The complete loop and its independence from epithelial injury have not been demonstrated in CRS or AR. Path A shows a possible route involving TL1A, CCL8, ILC2s, and Th2 cells; Path B shows an indirect neutrophil-recruitment route involving CXCL8/IL-8. Both routes may also be driven by parallel epithelial, stromal, microbial, and eicosanoid signals.

**Table 3:** Proposed TL1A-macrophage loop and its possible relationship to epithelial alarmins.

Characteristic	Traditional epithelial alarmins (IL-33, TSLP, IL-25)	Candidate TL1A-macrophage autocrine loop	References
Cellular Source	Primarily epithelial-derived, although release or induction can be context dependent	Proposed macrophage contribution; direct CRS/AR evidence limited	(54, 61, 62, 66)
Persistence of Signal	Transient (highly dependent on continuous epithelial damage)	Could be sustained by positive feedback; independence from epithelial injury is unconfirmed in sinonasal tissue	(54, 58, 63, 64, 67)
Scope of Action	Locally restricted innate initiating signals	Potentially broader myeloid/lymphoid effects, but upper-airway extent remains to be tested	(54, 59, 65, 68)
Relationship to TL1A	Can initiate or shape type 2 responses; the ordering relative to TL1A is context dependent	TL1A may act upstream, downstream, or in parallel. Cooperation with IL-33 is supported in a lower-airway model, whereas the ordering in CRS/AR remains untested	(26, 27, 44, 59, 61, 62)

### 4.1 Mechanism innovation: TL1A vs. traditional epithelial cytokines

To place the proposed TL1A pathway in context, it is important to distinguish its possible position relative to established epithelial alarmins. IL-33, TSLP, and IL-25 can be released or induced during epithelial stress and can initiate or shape type 2 responses (65, 66, 68). In CRSwNP, TNFSF11/RANKL- and TSLP-associated ILC2 biology also illustrates that type 2 inflammation can be supported by pathways that do not require TL1A (23, 24). Recent lower-airway work supports cooperation between epithelial TL1A and IL-33 rather than a simple one-directional hierarchy (21, 22). Thus, TL1A may act upstream, downstream, or in parallel depending on cell type, stimulus, and disease phase. The cooperation reported in lung models and the alternative TNFSF11/TSLP findings in CRSwNP should not be treated as proof of a fixed TL1A ordering in CRS or AR; the macrophage loop remains a candidate extension of this network (**Table 3**)(69–71).

### 4.2 Cellular distribution and signal transduction basis of DR3 receptors

The precise cellular distribution of DR3 is likely to shape any TL1A response (72). Evidence from mucosal and lower-airway systems supports DR3 expression and TL1A responsiveness in selected macrophage, ILC2, and Th2 or memory T-cell populations (23, 24, 30, 73). However, receptor abundance can vary with tissue, activation state, and disease endotype. Sinonasal epithelial cells and fibroblasts are major potential sources of CXCL8/IL-8 and remodeling mediators, but current CRS/AR studies do not establish whether these structural cells express functional DR3, respond directly to TL1A, or are affected only through secondary mediators released by immune cells. A transcript or protein signal alone would not demonstrate functional receptor activity. If structural cells are direct TL1A targets, they could provide an additional route to chemokine production, barrier alteration, or remodeling; if they are not, these effects would more plausibly be indirect. **Figure 5** therefore presents a simplified, non-exhaustive map of candidate receptor-positive compartments rather than a complete upper-airway receptor atlas (74).

**Figure 5:**
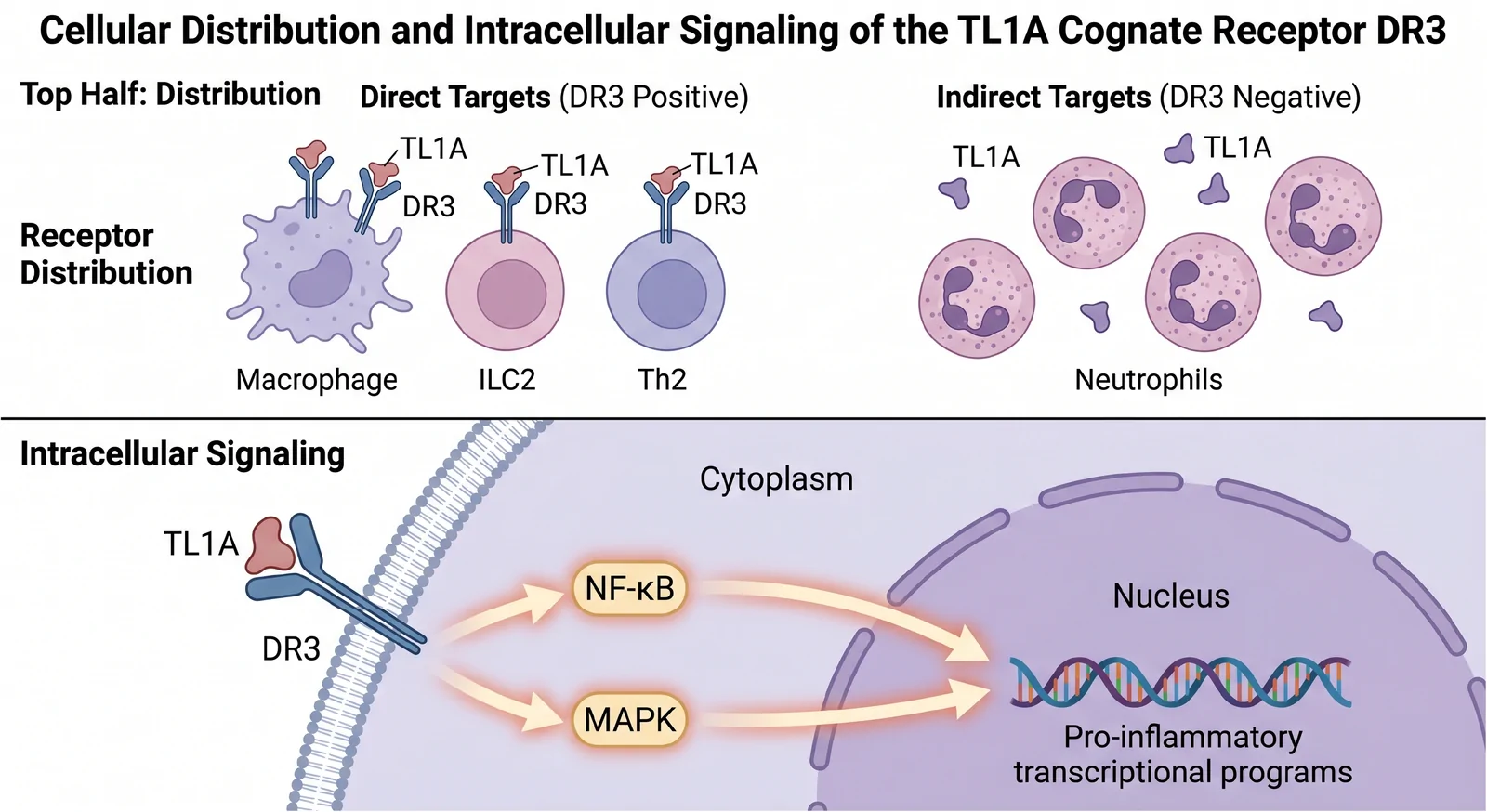
Candidate cellular distribution of DR3 and associated intracellular signaling pathways. (Top) The diagram presents macrophages, ILC2s, and Th2 or memory T-cell populations as candidate direct targets of TL1A/DR3 based mainly on mucosal and lower-airway evidence. Human CRS/AR data do not yet establish functional DR3 signaling in sinonasal epithelial cells or fibroblasts, although these structural cells may respond directly or indirectly and can produce CXCL8/IL-8 and remodeling mediators. Neutrophils are shown as primarily indirect targets because direct DR3 expression is generally limited. (Bottom) NF-kappaB and MAPK signaling are representative pathways described in relevant experimental contexts; the figure is simplified and non-exhaustive.

Neutrophils are generally considered to have little or no direct DR3 expression in the contexts summarized here (67). They should therefore be treated primarily as indirect targets of a TL1A-centered network, recruited through macrophage-, epithelial-, or stromal-cell-derived mediators such as CXCL8/IL-8. This distinction has therapeutic implications: inhibiting TL1A may reduce upstream relay activity if TL1A is validated as a driver, but it may not resolve established neutrophilic inflammation by itself. Downstream chemokine pathways, barrier defects, infection, and tissue remodeling may require separate evaluation (75).

### 4.3 Macrophages as both effectors and sources: the autocrine loop

If epithelial-derived TL1A engages local DR3-positive macrophages, these cells could undergo inflammatory reprogramming and become both effectors and additional sources of TL1A (76). A positive-feedback loop in which macrophage-derived TL1A reinforces macrophage activation is supported in selected experimental systems, but a complete epithelial-TL1A-to-macrophage-to-TL1A circuit has not been directly demonstrated in CRS or AR. The direction, magnitude, and persistence of this feedback may depend on ongoing epithelial injury and on other signals in the tissue (77).

### 4.4 Paracrine recruitment and inflammatory network expansion

Macrophages could also extend the response through paracrine signaling, but their status as the central hub in sinonasal inflammation remains a hypothesis (78). TL1A from macrophages may stimulate DR3-positive ILC2s, Th2 cells, or memory T-cell populations in some contexts, while CCL8 and other chemokines may shape recruitment and cellular positioning. The relative contribution of macrophage-derived TL1A versus epithelial, stromal, or circulating sources has not yet been resolved in CRS or AR (79, 80).

A possible route to mixed inflammation involves macrophage-derived CXCL8/IL-8 and related chemokines. The scoping review by Cergan et al. describes IL-8 as part of a molecular interplay involving epithelial, myeloid, and structural-cell programs in allergic rhinitis and CRSwNP, rather than as a macrophage-specific output (81). This distinction is important because increased CXCL8/IL-8 cannot, by itself, identify a TL1A-dependent pathway. The specific upstream relationship between TL1A/DR3 signaling and CXCL8/IL-8 production in sinonasal tissue is not established. TL1A may contribute to this relay, act in parallel with other drivers, or be secondary to established inflammation. Paired macrophage–epithelial or macrophage–fibroblast cultures, tissue explants, and TL1A/DR3 perturbation studies with measurements of CXCL8 release and neutrophil migration would help distinguish these possibilities (82–84).

In summary, an epithelial injury-associated response could evolve into a macrophage-mediated autocrine and paracrine network, but this remains a mechanistic model. Its central predictions are that TL1A-responsive macrophages can be identified in relevant sinonasal compartments, that they can produce biologically active TL1A, and that interrupting TL1A/DR3 changes downstream lymphoid and chemokine responses. The model also predicts that TL1A blockade alone may be insufficient when neutrophilia is sustained by CXCL8/IL-8, infection, barrier dysfunction, fibroblast remodeling, or other parallel pathways.

## 5 Hypothesized clinical endotype evolution: from type 2-dominant to mixed/neutrophilic inflammation

The TL1A-macrophage axis could contribute to clinical heterogeneity, but it is one candidate component of a network that also includes epithelial-barrier status, alarmins, eicosanoids, fibroblasts, microbes, and tissue-resident immune cells (85). This section maps the proposed TL1A relationships to clinical patterns while distinguishing mechanistic plausibility from direct evidence in CRS and AR. **Figure 6** visualizes this mapping as a hypothesis rather than a clinical staging system (86).

**Figure 6:**
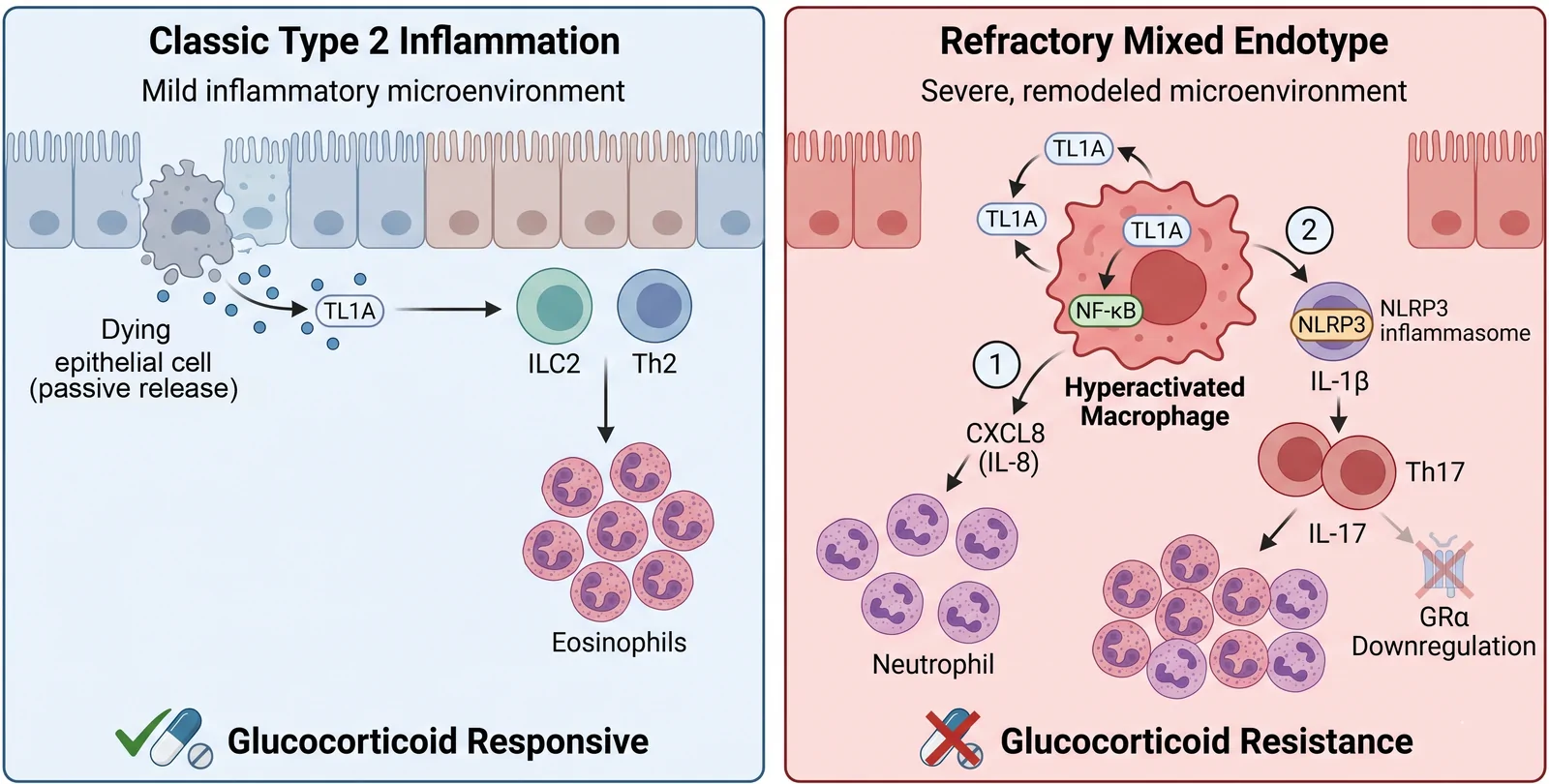
Hypothesized clinical-mechanistic mapping from type 2-dominant to mixed inflammation. (Left Panel) In type 2-dominant inflammation, TL1A/DR3 may contribute to ILC2 and Th2 activation alongside IL-33, TSLP, and IL-25. (Right Panel) In mixed or refractory disease, a macrophage-centered TL1A relay could intersect with CXCL8/IL-8, NLRP3/IL-1beta, Th17, stromal, microbial, and barrier pathways. This is a conceptual mapping rather than a clinical staging scheme; TL1A alone is not presumed sufficient to cause neutrophilia or steroid resistance.

### 5.1 Type 2-dominant inflammation: the candidate TL1A-ILC2 axis

In type 2-dominant phenotypes, such as many cases of AR and some treatment-naive CRSwNP (87), the mucosa may be enriched for ILC2s, Th2 cells, eosinophils, and type 2 cytokines (88). TL1A/DR3 signaling is a plausible contributor because ILC2 and memory T-cell responses to TL1A are documented in other airway or mucosal contexts (23, 30, 73). Nevertheless, local TL1A abundance and the relative importance of TL1A compared with IL-33, TSLP, and IL-25 have not been defined in these upper-airway phenotypes. Conventional type 2 biologics may be effective when the dominant circuit is eosinophilic, but a TL1A contribution should be tested rather than assumed (89–92).

### 5.2 Heterogeneous endotypes and refractory rhinosinusitis

The clinical reality of CRS frequently deviates from a pure type 2 pattern (14). Difficult-to-treat or recurrent disease can show mixed eosinophilic and neutrophilic infiltrates, tissue remodeling, altered barrier function, and reduced responsiveness to corticosteroids (93). These features are compatible with a macrophage-chemokine relay, but they can also arise from epithelial injury, infection or microbial dysbiosis, eicosanoid imbalance, fibroblast activation, or type 17 pathways. A mixed phenotype should therefore be viewed as a convergent outcome, not as proof of a TL1A-dependent sequence (94, 95).

Single-cell studies of CRS and nasal polyps have revealed substantial heterogeneity in epithelial, myeloid, lymphoid, and stromal compartments (96, 97). They motivate examination of TL1A and DR3 at cell-state resolution, but the available sinonasal evidence does not by itself establish a TL1A-positive/DR3-positive macrophage circuit, a causal TL1A-to-neutrophil pathway, or a complete macrophage feedback loop in severe CRS. Findings from lower-airway and intestinal models provide mechanistic hypotheses, not direct proof of the same architecture in the upper airway. In addition, TL1A is not uniformly increased across tissues or cell states: reduced monocyte and macrophage TNFSF15/TL1A expression has been reported in an inflammatory bowel disease context (98). This finding may reflect disease- and cell-state-specific biology, but its precise explanation is unresolved and it argues against assuming that all inflammatory myeloid populations increase TL1A. Together with variable DR3 expression across tissues, these observations support endotype- and compartment-specific measurement rather than a universal TL1A signature (99–101).

One possible mechanism involves an NF-kappaB/CXCL8 chemotactic pathway. Macrophage or structural-cell activation can increase CXCL8/IL-8, a mediator linked to neutrophil recruitment and to broader inflammatory interplay in AR and CRSwNP (81). TL1A/DR3 signaling could participate in this pathway in selected cell states, but direct TL1A-dependent induction of CXCL8 in sinonasal tissue has not been demonstrated. Other upstream signals may be equally or more important.

A second, putative route involves the NLRP3/IL-1beta-Th17 axis (102). TL1A/DR3 ligation can provide inflammatory context for myeloid activation in some experimental systems, and NLRP3 or IL-1beta pathways are relevant to neutrophilic and type 17 inflammation. However, direct TL1A-dependent NLRP3 activation in human sinonasal macrophages, its relationship to epithelial injury, and its effect on steroid responsiveness remain unconfirmed. The model should therefore be tested against alternative inflammasome triggers and infection-related signals (103–105).

### 5.3 Proposed clinical-mechanistic mapping

To provide a cohesive translational framework, the following mapping treats the transition from type 2-dominant AR or CRS to mixed, refractory disease as a set of testable associations rather than a fixed TL1A trajectory. mTL1A may support localized contact-dependent signaling; sTL1A may broaden signaling if shedding occurs; and macrophage-derived CXCL8/IL-8, IL-1beta, or other mediators may connect receptor-positive and receptor-negative compartments. Each step may also be driven or modified by epithelial-barrier dysfunction, eicosanoids, fibroblasts, microbiome changes, and other alarmins (106).

In summary, the TL1A-macrophage axis is a candidate bridge between type 2-dominant and mixed upper-airway inflammation, not a demonstrated determinant of the entire endotypic spectrum. The value of this framework lies in its predictions: TL1A/DR3 activity should track with specific cell states or tissue compartments, local mTL1A and sTL1A should be measurable if the proposed morphological shift occurs, and pharmacologic or genetic interruption should alter macrophage, lymphoid, and CXCL8/IL-8 responses in a context-dependent manner.

## 6 Discussion and translational medicine perspectives

The united-airway concept supports searching for mechanisms that can operate across respiratory compartments, but it does not imply that a pathway established in the lower airway or intestine will have the same source, receptor distribution, or therapeutic value in CRS or AR. TL1A is a plausible candidate within a broader network that includes epithelial-barrier dysfunction, IL-33/TSLP/IL-25 signaling, eicosanoid imbalance, fibroblast remodeling, microbial dysbiosis, and tissue-resident myeloid programs (14–16). Its potential advantage would need to be demonstrated experimentally in the specific endotypes and compartments in which it is active; **Table 4** summarizes these potential advantages alongside the main translational uncertainties (107, 114).

**Table 4:** Potential advantages and translational uncertainties of targeting the TL1A axis in united-airway disease.

Comparison metrics	Traditional biologics (e.g., Anti-IL-4Rα/Anti-IL-5)	Potential TL1A-targeted therapies	References
Endotype Coverage	Limited to isolated type 2 eosinophilic responses	Potentially broader coverage of mixed endotypes; clinical benefit in CRS/AR remains untested	(107, 108)
Mechanism of Action	Block single downstream cytokine signaling pathways	Could interrupt TL1A/DR3 signaling and a putative macrophage feedback loop; direct sinonasal mechanism remains unconfirmed	(107, 109, 110)
Efficacy in Refractory CRS	Suboptimal; fail to dismantle the broader, macrophage-driven inflammatory networks	Unproven; requires dedicated preclinical and clinical evaluation	(111, 112)
Glucocorticoid Refractoriness	Demonstrates limited or insufficient improvement	May influence downstream inflammatory signals, but reversal of steroid resistance has not been established	(112)
Delivery and safety	Established administration routes and safety profiles vary by agent	Systemic versus topical delivery, sinonasal penetration, infection risk, and epithelial-repair effects require study	(107, 108, 110, 113)

Targeting TL1A could be relevant for refractory rhinosinusitis with mixed inflammation if a TL1A-dependent macrophage relay is confirmed. Blocking TL1A/DR3 might reduce selected lymphoid and upstream myeloid signals, but it may not eliminate established neutrophilia, infection, barrier failure, fibroblast remodeling, or CXCL8/IL-8-driven recruitment. There is currently no direct clinical evidence that anti-TL1A therapy improves CRS or AR, so claims of superior efficacy or reversal of steroid resistance should remain prospective.

Delivery is a separate translational issue. Systemic anti-TL1A administration may provide sustained exposure to submucosal and extra-sinus compartments, but it would also increase systemic immunologic and infection-related exposure. Topical or intranasal delivery could increase local concentrations and reduce systemic exposure, yet nasal sprays or other local formulations may be limited by mucus, mucociliary clearance, ostial obstruction, sinus anatomy, and poor penetration into polyp tissue. Local treatment would also require assessment of epithelial repair, mucociliary function, immunogenicity, and infection risk. Whether local dosing can reach DR3-positive immune and structural-cell compartments without compromising mucosal defense is unknown; comparative pharmacokinetic, tissue-penetration, and safety studies are needed before repurposing.

Clinical development of anti-TL1A agents in IBD demonstrates that the pathway is druggable and provides useful safety and pharmacodynamic precedents (108, 109, 111). Shared mucosal biology makes repurposing an interesting hypothesis, but IBD efficacy cannot be taken as evidence of CRS or AR efficacy. Upper-airway trials would need to define the target endotype, demonstrate local target engagement, and monitor infection risk, epithelial repair, and effects on coexisting type 2, type 17, and neutrophilic pathways (110, 112, 113, 115–117).

Looking forward, the structural biology of TL1A may support personalized investigation in otolaryngology, but mTL1A and sTL1A should be considered potential biomarkers rather than validated prognostic markers (25). Priority studies should quantify both forms in paired nasal polyp tissue, inferior turbinate mucosa, nasal lavage fluid, blood, and other relevant clinical specimens; distinguish membrane-associated protein from soluble protein; map DR3 across immune, epithelial, fibroblast, and endothelial compartments; and test whether values predict endotype, exacerbation, treatment response, or local target engagement. These studies should also measure IL-33, TSLP, IL-25, CXCL8/IL-8, and relevant infection or remodeling signals so that TL1A is evaluated within, rather than separated from, the broader sinonasal network. Contradictory tissue-level observations, including reduced TL1A expression in some myeloid contexts (98), make cell-state and compartment resolution essential. Patient-derived sinonasal cultures, organoids, tissue explants, and carefully selected animal models can then test whether TL1A blockade changes the proposed macrophage, CXCL8/IL-8, and remodeling circuits (26, 27).
